# Protective Effect of Aqueous Extract from the Leaves of *Justicia tranquebariesis* against Thioacetamide-Induced Oxidative Stress and Hepatic Fibrosis in Rats

**DOI:** 10.3390/antiox7070078

**Published:** 2018-06-22

**Authors:** Kumeshini Sukalingam, Kumar Ganesan, Baojun Xu

**Affiliations:** 1 Faculty of Medicine, International Medical School, Management and Science University, Shah Alam 40100, Malaysia; meshni_anat@yahoo.com.my (K.S.); drbiokumar@yahoo.com (K.G.); 2 Food Science and Technology Program, Beijing Normal University–Hong Kong Baptist University United International College, Zhuhai 519087, China

**Keywords:** *Justicia tranquebariesis*, TAA, oxidative stress, hepatic fibrosis, hepatoprotection

## Abstract

The present study aims to examine the protective effect of *Justicia tranquebariesis* on thioacetamide (TAA)-induced oxidative stress and hepatic fibrosis. Male Wister albino rats (150–200 g) were divided into five groups. Group 1 was normal control. Group 2 was *J. tranquebariensis* (400 mg/kg bw/p.o.)-treated control. Group 3 was TAA (100 mg/kg bw/s.c.)-treated control. Groups 4 and 5 were orally administered with the leaf extract of *J. tranquebariensis* (400 mg/kg bw) and silymarin (50 mg/kg bw) daily for 10 days with a subsequent administration of a single dose of TAA (100 mg/kg/s.c.). Blood and livers were collected and assayed for various antioxidant enzymes (SOD, CAT, GPx, GST, GSH, and GR). Treatment with *J. tranquebariensis* significantly reduced liver TBARS and enhanced the activities of antioxidant enzymes in TAA-induced fibrosis rats. Concurrently, pretreatment with *J. tranquebariensis* significantly reduced the elevated liver markers (AST, ALT, ALP, GGT, and TB) in the blood. In addition, *J. tranquebariensis-* and silymarin- administered rats demonstrated the restoration of normal liver histology and reduction in fibronectin and collagen deposition. Based on these findings, *J. tranquebariensis* has potent liver protective functions and can alleviate thioacetamide-induced oxidative stress, hepatic fibrosis and possible engross mechanisms connected to antioxidant potential.

## 1. Introduction

Liver fibrosis is a scarring mechanism of the liver related to prominent accretion of an extracellular matrix. Without efficient treatment at an early stage, reversible liver fibrosis develops into irreversible cirrhosis leading to liver failure, portal hypertension and the need for liver transplantation in many cases [1]. These progressive scarring insults result in liver cirrhosis, which is the foremost health burden leading to death worldwide. At present, no known therapeutic approach is widely available, except for the removal of the fibrogenic stimuli. However, in vivo and clinical studies have established that liver fibrosis and cirrhosis are reversible to heal, but still inadequate for widely used known treatments [2,3,4,5]. The liver fibrosis is usually caused by diverse chronic insults including, chemicals, parasitic infections, alcohol, viral hepatitis B and C, and autoimmune hepatitis. Due to the worldwide occurrence of these insults, liver fibrosis is widespread and is related to liver linked morbidity and mortality [6]. Chronic liver injury normally causes progressive liver fibrosis distinguished by alterations of both quality and quantity of hepatic extracellular matrix proteins including collagen, which occurs in most types of end-stage liver diseases [7]. Furthermore, this chronic liver injury is triggered by an inappropriate balance between the generation and destruction of ROS, which results in hepatocyte damage and abnormal tissue injury [8]. Thioacetamide (TAA) is an organosulphur. It is a chemical, which is extensively used as a fungicide in various industries including textile dyes [7]. Presently, it is considered as a carcinogen. It is rapidly metabolized into free radical derivatives such as TAA sulfoxide and TAA-S-S-dioxide, which leads to lipid peroxidation, and eventually culminates in centrilobular damage and liver injury [9]. Earlier studies have also demonstrated that the exposure to TAA caused liver injury, fibrosis, steatosis, and cirrhosis in experimental animals [1,9]. Hence, TAA is recognized as a model of liver fibrosis in rats. Currently, the widely used treatment of liver fibrosis and cirrhosis is inadequate; and there is no effectively widely used therapy that can prevent the development of hepatic diseases. Although recently developed drugs have been used to heal liver diseases, often these drugs have numerous side-effects. There is, thus, an urgently requirement for alternative remedies or drugs for the treatment of chronic liver disorders to replace present drugs of uncertain safety and effectiveness [10]. For this intention, herbal constituents and dietary supplements have potential as alternative medicines for the treatment of chronic liver diseases and related metabolic derailments [11].

*Justicia tranquebariensis* L. (Family: *Acanthaceae*), a common shrub is broadly scattered in various regions of India, Malaysia and Sri Lanka. The fresh leaves are coolant and aperient, generally used in jaundice, liver diseases, and smallpox [12]. Bruised leaves are applied to contusions, diaphoretic, diuretic, and rheumatism and used as antidotes for snake bite [13]. The juice of the leaves is used as an expectorant in cold, cough, nasal disorders, whereas, the paste of the leaves is applied externally to treat skin diseases, swelling and pain. The root could also be made into a paste to treat toothaches. Phytochemical studies of *J. tranquebariensis* revealed that the leaves contain adequate quantities of phytosterols, flavonoids, glycosides, triterpenoids, alkaloids, saponins, and tannins [14,15]. Aerial parts of the plant contain lignans including aryl tetralin, β-cubebin, lariciresinol, isolariciresinol, lyoniresinol and medioresinol [16]. In addition, the alcoholic extract yielded 7,22-ergostadienol, 28-isofucosterol, β-sitosterol-3-*O*-glucoside, brassicasterol, campesterol, stigmasterol, sitosterol, and spinasterol [12]. Furthermore, the plant has various pharmacological potentials including free radical scavenging, anti-inflammatory [14], antipyretic [15], antimicrobial [17], and antihepatotoxicity [18] potentials. Based on previous literature, no information was available on the hepatoprotective and antioxidant effect of the *J. tranquebariensis* leaf extract on liver fibrosis. Thus, this current investigation aimed to observe the antifibrotic and hepatoprotective effect of *J. tranquebariesis* on TAA-intoxicated rats.

## 2. Materials and Methods 

### 2.1. Plant Material and Preparation

**Figure 1:**
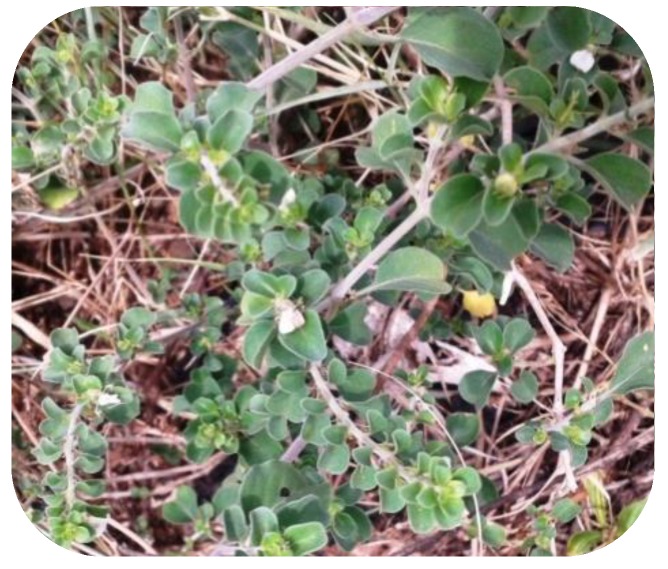
Leaves of *Justicia tranquebariensis* L.

The fresh leaves of *Justicia tranquebariensis* L. (Figure 1) were collected during February–March 2014 from Shah Alam, Selangor, Malaysia and were authenticated by a taxonomist, Sujit Sarker, Department of Pharmacognosy and comparison with reference materials conserved in the Herbarium and voucher specimens were kept in the institution. Coarse powdered leaves (1000 g) were subjected to 2 L of distilled water, and extraction was maintained with regular stirring for 8 h. The extract was centrifuged, and the supernatants were evaporated using a vacuum rotary evaporator and residues were kept in refrigeration for further use (yield: 180 g/1000 g). 

### 2.2. Animals

Male Wister albino rats (150–200 g) were acquired from the central animal house in Management and Science University, Shah Alam, Selangor, Malaysia. Animals were kept in animal cages under standard lab conditions (12 h alternating day and night cycles, rooms were air-conditioning at 25–28 °C). Rats were adapted to the laboratory settings for a week prior to the initiation of experimental treatments. Rats were supplied with free access to standard pellet food and water. The experimental studies were carried out based on the ethical approval and the protocol was permitted by the Institutional Ethical Committee of Management and Science University, Malaysia (Reg no: 12/2011/CPCSEA, proposal no: 75).

### 2.3. Chemicals

TAA, thiobarbituric acid (TBA), 1-chloro-2,4-dinitrobenzene (CDNB), and nicotinamide adenine dinucleotide hydrogen phosphate (NADPH) were procured from Sigma-Aldrich Co., St. Louis, MO, USA. Reagents and chemicals used in the studies were of the analytical grade.

### 2.4. Dose Determination

A preliminary study was carried out to validate the optimal dose of plant extract by examining serum hepatic marker enzymes in TAA-intoxicated rats. Administration of aqueous extract from the leaves of *J. tranquebariensis* was given at various doses of 100, 200, 400, 800 mg/kg bw to different groups of rats. Among the doses, the 400 mg/kg bw showed more effectiveness than the other doses. Hence 400 mg/kg bw was used in this investigation. The dosage of TAA (100 mg/kg bw) and standard drug silymarin (50 mg/kg bw) used in the present study were chosen according to a previous study [19].

### 2.5. Experimental Design

Following laboratory adaptation, animals were randomly divided into five groups, each group comprising of six rats. All rats were kept fasting for 24 h before the experiment. Group 1 (normal control): Rats of this group received 5 mL of distilled water/kg bw. Group 2 (*J. tranquebariensis* control): Rats of this group were pretreated with *J. tranquebariensis* (400 mg/kg bw p.o./day) for 10 days. Group 3 (toxic control): Rats of this group were treated with a single dose of TAA (100 mg/kg bw/s.c.) on the 10th day. Group 4 (*J. tranquebariensis* plus TAA): Rats of this group were pretreated with *J. tranquebariensis* (400 mg/kg bw p.o./day) for 10 days and subsequent administration of a single dosage of TAA (100 mg/kg/s.c.). Group 5 (Silymarin plus TAA): Rats of this group were pretreated with silymarin (50 mg/kg bw/p.o./day) for 10 days followed by a single dose subcutaneous injection of TAA (100 mg/kg). TAA hepatotoxicity induction was followed our previous investigation [20]. All animals were deprived of food overnight and sacrificed by using anesthesia followed by cervical dislocation. Blood was collected from each respective group for the assay of biochemical parameters. The liver was immediately isolated, immersed in cold saline and weighed. A piece of one gm of liver from each rat was taken and homogenized to make liver homogenate, which were then centrifuged and the supernatant obtained was subjected to tissue biochemical parameters.

### 2.6. Biochemical Parameters

The activities of serum ALP, ALT, AST, GGT and TB were quantified by using commercial kits (Premier Diagnostics Sdn Bhd, Shah Alam, Malaysia). In the liver tissue homogenates, LPO was calculated using TBARS based on the method of Ohkawa et al. [21]. SOD and CAT were measured in liver tissue homogenate based the method of Marklund et al. [22] and Sinha [23] respectively. Activities of the respective enzymes were assayed by the methods as GR by Bellomo et al. [24], GPx by Rotruck et al. [25], GST by Habig et al. [26], and total GSH by Moron et al. [27]. The quantification of protein was done by the method of Lowry et al. [28].

### 2.7. Histological Investigations

After sacrificing the animals, the livers were quickly removed and preserved in 10% formosaline and processed for paraffin embedding following the standard micro techniques. Sections (5 µm thick) of liver tissues stained with hematoxylin and eosin (H&E) were evaluated for histopathological under a light microscope. 

### 2.8. Statistical Analysis

All data obtained in the study were expressed as mean ± standard deviation (S.D.). The data of the groups were statistically done using one-way analysis of variance (ANOVA) and the individual comparison was obtained by Duncan’s Multiple Range Test (DMRT) by the SPSS software for Windows Version 20.0 (IBM Corp. Armonk, New York, NY, USA). A value of *p* < 0.05 was considered to indicate a significant difference between groups.

## 3. Results

### 3.1. Effects of J. tranquebariensis on Hepatic TBARS and Antioxidant Enzymes

**Figure 2:**
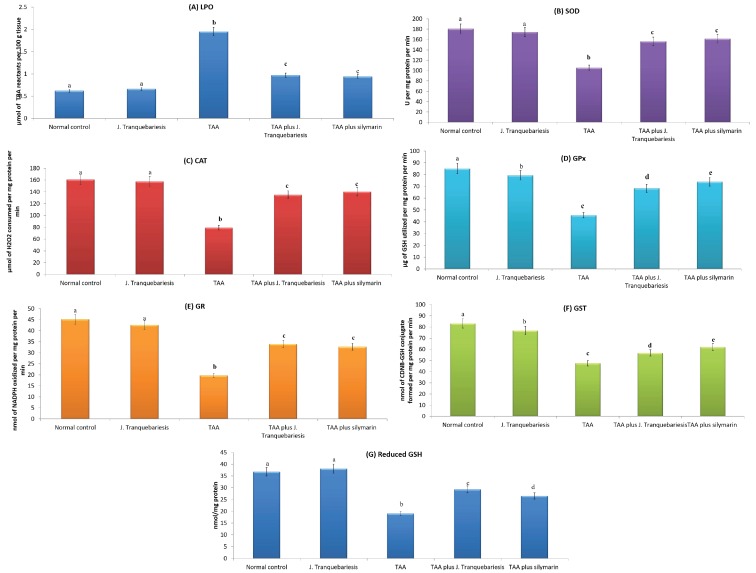
(**A**–**G**) Effects of *Justicia tranquebariesis* L. on liver lipid peroxidation and antioxidant enzyme activities in TAA intoxicated rats. Values are expressed as mean ± S.D. for six animals in each group. Values not sharing a common superscript (a–e) differ significantly. Letter “a” is significant to b, c, d, and e; likewise the letter “b” is significant to a, c, d and e. LPO—lipid peroxidation; TBA—thiobarbituric acid; SOD—superoxide dismutase; CAT—catalase; GPx—glutathione peroxidase; GR—glutathione reductase; NADPH—nicotinamide dinucleotide phosphatase; GST—glutathione transferase; CDNB—1-chloro-2,4-dinitrobenzene; GSH—glutathione.

The levels of tissue TBARS formation and the activities of antioxidant enzymes SOD, CAT, GPx, GR, GST and total reduced GSH in the liver of normal control and experimental rats are demonstrated in Figure 2A–G. In TAA (100 mg/kg bw)-treated rats, all antioxidant enzymes SOD, CAT, GPx, GR, GST and total GSH were found to be significantly decreased (*p* < 0.05), whereas tissue TBARS level significantly increased (*p* < 0.05) when compared with the normal control group. However, pretreatment administration of *J. tranquebariensis* (400 mg/kg bw) and silymarin (50 mg/kg bw) in TAA-induced rats have significantly altered to the above changes by regulating the TBARS level which subsequently increased those antioxidant enzymes.

### 3.2. Effects of J. tranquebariensis on Liver Marker Enzymes and Total Bilirubin

**Figure 3:**
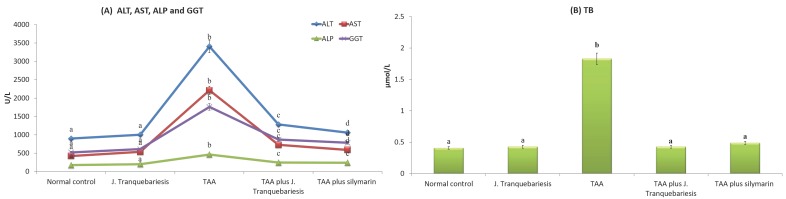
(**A**,**B**) Effects of *Justicia tranquebariesis* L. on hepatic markers and TB in TAA intoxicated rats. Values are expressed as mean ± S.D. for six animals in each group. Values not sharing a common superscript (a–d) differ significantly. Letter “a” is significant to b, c, and d; likewise a letter “b” is significant to a, c, and d. TB—total bilirubin; ALT—alanine aminotransferase; AST—aspartate aminotransferase; ALP—alkaline phosphatase; GGT—gamma-glutamyltransferase.

The activities of liver marker enzymes such as ALT, AST, ALP, GGT and the content of TB in the serum of control and experimental groups are shown in Figure 3A,B. The activities of ALT, AST, ALP, GGT and the content of total bilirubin in serum has significantly increased (*p* < 0.05) in TAA-intoxicated rats when compared with the normal control group. However, pretreatment of *J. tranquebariensis* (400 mg/kg bw) significantly decreased (*p* < 0.05) the above liver marker enzymes and total bilirubin levels observed in TAA-intoxicated rats.

### 3.3. Histological Observation

**Figure 4:**
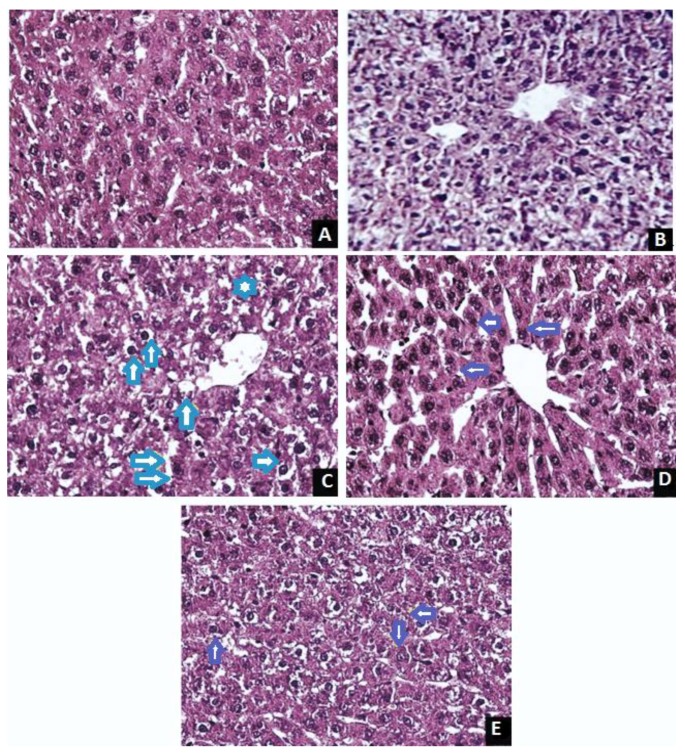
Micrographs showing the effect of *Justicia tranquebariensis* on TAA-induced hepatic fibrosis in rats. (**A**) Section of normal control rats showing the histological appearance of hepatocytes with prominent nuclei and cytoplasm (H&E. 400×); (**B**) Section of *J. tranquebariensis* (400 mg/kg bw/p.o.) treated control rats showing the histological appearance of hepatocytes with prominent nuclei and cytoplasm (H&E. 400×); (**C**) Section of TAA (100 mg/kg bw/s.c.) treated control showing fatty degeneration of some hepatocytes, Kupfer cells characterized by cell swelling, the replacement of the cytoplasm with a clear fluid and a centrally located nucleus (↑↑ blue), loss of cell boundaries, hepatic necrosis, collagen and fibronectin deposition and inflammatory cell infiltration (H&E. 400×); (**D**) Section of TAA (100 mg/kg bw/s.c.) plus *J. tranquebariensis* (400 mg/kg bw/p.o.) treated rats showing regenerated cells and the almost normal architecture of the liver (↑ violet) with a decrease in collagen and fibronectin deposition (H&E. 400×); (**E**) Section of TAA (100 mg/kg bw/s.c.) plus silymarin (50 mg/kg bw)-treated rats showing regenerated cells and the almost normal architecture of the liver (↑ violet) with decrease in collagen and fibronectin deposition (H&E. 400×).

The histology of normal control, TAA control, *J. tranquebariensis* and silymarin-treated rats are exhibited in Figure 4. The liver sections of normal control groups showed the normal architecture of hepatocytes with prominent nucleus, well-preserved cytoplasm and visible central vein. TAA-administered animals showed hepatocytes with severe toxicity described by extensive necrosis, collagen and fibronectin deposition with sinusoidal spaces, and a central venule. Tissue showed severe cell swelling, vacuolar degeneration, loss of cell boundaries and the replacement of the cytoplasm with fluid and a centrally positioned nucleus. The livers of the rats pretreated with *J. tranquebariensis* (400 mg/kg bw) and silymarin (50 mg/kg bw) showed a normal lobular pattern with sinusoidal spaces, moderate swelling, and restoration of normal liver histology and thereby a decrease in collagen and fibronectin deposition.

## 4. Discussion

In the present study, we investigated the effect of an aqueous extract from the leaves of *J. tranquebariensis* on TAA-induced liver fibrosis in rats. A noticeable indication of liver injury is the release of cytoplasmic cellular enzymes into the blood due to the obstructions caused by chemicals in the transport functions of liver cells [8]. The increases in serum enzymes are markers for liver injury/damage. A significant increase in ALT, AST, and GGT can be considered as marker enzymes of liver damage. Serum transaminases were reduced and GGT returned to normal by pretreatment of *J. tranquebariensis* with a healing of hepatic parenchyma and regeneration of hepatocytes [29,30]. The concentrations of the ALP and TB have also been used as hepatic markers in chemically induced hepatic injury. In the toxicity studies, about 80% of serum ALP levels have been found to be elevated in rodents [31,32]. Pretreatment of *J. tranquebariensis* prevented the TAA toxicity effect on ALP activity in serum. In addition, the normalization of serum TB levels is done by the administration of *J. tranquebariensis*, which shows an indication of the normal functions of the hepatocyte.

GSH in the cytosolic pool consists of 85% hepatocellular GSH and 15% mitochondrial GSH. The reductions of hepatic GSH provide valuable evidence of the defensive role of GSH against noxious foreign materials [33]. Therefore, GSH is considered as an endogenous defensive mediator against various drugs [34]. A large dose of TAA causes hepatic GSH depletion because the excess TAA derivative reacts rapidly with GSH, which exacerbates oxidative stress in conjunction with mitochondrial dysfunction [8,35]. In the present study, pretreatment of *J. tranquebariensis* clearly enhanced GSH levels and facilitated the rapid and efficient consumption of ROS generation in TAA-intoxicated rats. GST is a soluble enzyme located in the cytosol, which plays a significant function in the detoxification of xenobiotics [8]. It increases the solubility of hydrophobic substances and metabolizes toxic compounds to non-toxic ones, which means they have an increasing protective activity of the liver [36]. The increased hepatic GST activity induced by *J. tranquebariensis* can, therefore, reduce TAA hepatotoxicity. There was a decrease in GPx activity in animals administered with TAA, which could be due to the higher production of toxicity. In the presence of *J. tranquebariensis*, GPx levels were restored back to control levels. The increase in hepatic GR activities was shown in *J. tranquebariensis* administered rats, as compared with the liver of TAA-induced rats. Elevated levels of SOD and CAT are insightful indexes into liver damage as they eradicate ROS, thereby reducing the harmful effects. On the contrary, an earlier report showed that TAA induction raises the TBARS levels, which instigated to reduce the levels of SOD and CAT [1]. However, pretreatment with *J. tranquebariensis* and silymarin significantly decreased TBARS and elevated antioxidant enzymes in TAA-induced rats. Earlier studies have also suggested that *J. tranquebariensis* has strong antioxidant potential [18] and prevents ROS and/or RNS-mediated tissue damage [17].

The degree of protection was maximally observed in silymarin (50 mg/kg bw/p.o)-treated groups when compared with *J. tranquebariensis* (400 mg/kg bw). This degree of hepatoprotection could be based on its various properties of anti-oxidation, inhibition of lipid peroxidation, regeneration of intracellular glutathione content, protein synthesis and improvement of liver regeneration from hepatocellular necrosis [37,38,39,40,41,42,43]. Furthermore, it stabilizes cellular membranes and regulates membrane permeability that inhibits toxins entry into liver cells. Silymarin inhibits fibrogenesis in the liver by inhibition of stellate cell proliferation and its further transformation into myofibroblasts [44,45,46,47].

In the present study, we induced hepatic fibrosis by subcutaneous injection of a single dose of TAA. TAA is normally metabolized by CYP2E1 releasing ROS, which attacks DNA, lipid, and protein [48]. This may cause centrilobular necrosis, dilated sinusoidal spaces, collagen and fibronectin deposition and diffuse hyaline necrosis with blood pooling in sinusoidal spaces, accompanied by a marked attenuation of a normal liver function. The results of the TAA group were in agreement with other studies [1,2]. However, the histopathological patterns of the livers of rats pretreated with *J. tranquebariensis* showed a normal lobular pattern with minimal pooling of blood in the sinusoidal spaces, moderate swelling, condense collagen and fibronectin deposition and re-establishment of liver architecture to normal. In conclusion, the result of this study revealed that pretreatment of *J. tranquebariensis* has a strong hepato-protective effect on TAA-induced liver oxidative stress and liver fibrosis in rats owing to its strong antioxidant properties. However, further evidence is needed to establish the possible mechanisms of the hepatoprotection in order to widely use *J. tranquebariensis* as an antihepatotoxic agent.

## Abbreviations

ALP	alkaline phosphatase
ALT	alanine transaminase
ANOVA	one-way analysis of variance
AST	aspartate transaminase
bw	body weight
CAT	catalase
CDNB	1-chloro-2,4-dinitrobenzene
DMART	Duncan’s Multiple Range Test
GGT	gamma glutamyl transpeptidase
GPx	glutathione peroxidase
GR	glutathione reductase
GSH	reduced glutathione
GST	glutathione s-transferase
H&E	hematoxylin and eosin
Kg	kilogram
LPO	lipid peroxidation
mg	milligram
NADPH	nicotinamide adenine dinucleotide hydrogen phosphate
ROS	reactive oxygen species
s.c.	subcutaneous
S.D.	standard deviation
SOD	superoxide dismutase
SPSS	Statistical Package for the Social Sciences
TAA	thioacetamide
TB	total bilirubin
TBA	thiobarbituric acid
TBARS	thiobarbituric acid reactive substances
RNS	reactive nitrogen species
CYP2E1	cytochrome P450 2E1
